# Dental Cavity Grading: Comparing Algorithm Reliability and Agreement with Expert Evaluation

**DOI:** 10.1155/2024/3965641

**Published:** 2024-08-10

**Authors:** Abubaker Qutieshat, Abdurahman Salem, Melina N. Kyranides

**Affiliations:** ^1^ Adult Restorative Dentistry Oman Dental College, Muscat, Oman; ^2^ Restorative Dentistry Dental School University of Dundee, Dundee, UK; ^3^ Dental Technology School of Health and Society University of Bolton, Bolton, UK; ^4^ Clinical and Health Psychology School of Health in Social Science Medical School The University of Edinburgh, Edinburgh, UK

## Abstract

**Aim:**

The current study introduces a novel, algorithm-based software developed to objectively evaluate dental cavity preparations. The software aims to provide an alternative or complement to traditional, subjective assessment methods used in operative dentistry education.

**Materials and Methods:**

The software was tested on cavity preparations carried out by 70 participants on artificial molar teeth. These cavities were also independently assessed by an experienced academic panel. The software, using 3D imaging, calculated cavity dimensions and assigned an error score based on deviation from ideal measurements. Statistical analyses included sensitivity, specificity, positive predictive value, negative predictive value, Cohen's kappa, the intraclass correlation coefficient (ICC3k), Spearman's rho, Kendall's tau correlation coefficients, and a confusion matrix.

**Result:**

The software demonstrated a high degree of accuracy and agreement with the panel assessments. The average software and panel scores were 64.1 and 60.91, respectively. Sensitivity (0.98) was high, specificity (0.55) was moderate, and the ICC3k value (0.857) indicated a strong agreement between the software and the panel. Further, Spearman's rho (0.73) and Kendall's tau (0.56) suggested a strong correlation between the two grading methods.

**Conclusion:**

The results support the algorithm-based software as a valid and reliable tool for dental cavity preparation assessments. The software's potential use in dental education is promising, though future research is necessary to validate and optimize this technology for wider application.

## 1. Introduction

Dental education traditionally relies on hands-on training and practical assessments to evaluate students' skills and competence in various procedures. One critical aspect of dental education is the preparation of dental cavities, which demands a high degree of precision and accuracy to ensure successful restorations. A panel of experienced dental professionals typically carries out the assessment of students' performance in cavity preparation. They evaluate the quality of the work based on various criteria, such as the cavity's shape, depth, and preservation of the tooth structure.

However, the conventional assessment method involving an expert panel can be time-consuming, subjective, and potentially influenced by human error or bias [1, 2, 3]. Additionally, the growing number of dental students and the demand for more efficient and objective assessment methods have prompted investigations into alternative approaches, such as technology-aided assessment systems [4, 5, 6, 7, 8, 9]. These tools have the potential to provide more objective, accurate, and efficient evaluations than traditional panel-based assessments. Moreover, the use of technology-aided assessment systems can standardize the assessment process, enhance student feedback, and facilitate the identification of areas for improvement [10, 11, 12].

The aim of this study was to compare the efficacy of our specially developed open-source algorithm software with that of traditional expert panel evaluations in assessing dental cavity preparations. The null hypothesis proposed was that there would be no significant difference in the assessment results produced by the algorithm-based software and the expert panel.

In this study, we developed a specially programed open-source algorithm software, available at https://cephcad.com/dentalign/, for evaluating dental cavity preparations. This software's performance was compared with the assessments made by a panel of experienced academics who are specialists in restorative dentistry.

The findings from this study have the potential to contribute significantly to the expanding body of literature on technology's role in dental education, providing valuable insights into the benefits and limitations of implementing software applications for assessing dental cavity preparations. Ultimately, these results may equip dental educators and institutions with crucial knowledge for making informed decisions on adopting technology-enhanced assessment methods, thus enhancing the overall quality of dental education.

## 2. Methodology

This study has been approved by the Institutional Review Board under protocol number ODC-AE-2022-170, ensuring strict adherence to ethical guidelines for research involving human subjects. A cohort of 70 fourth-year dental students participated in this study. Participants were asked to prepare a cavity, a common exercise in operative dentistry, on an artificial mandibular third molar tooth (Nissin Dental Prod. Inc) in the traditional phantom head. The students were provided with precise instructions following ideal amalgam cavity preparation criteria. Regarding the outline, the participants were tasked with preparing the mesial and distal grooves, including the mesial, central, and distal pits. Clear specifications for ideal depth and width measurements were provided, with a depth of 1.50 mm and a width of 1.25 mm. While extending the cavity into the secondary and developmental grooves was optional, fulfilling the cavity convergence criterion was not requested. All participants used the same preparation bur (no. 330 bur, Komet) and were equipped with a standardized Williams graduated periodontal probe (Hu-Friedy).

### 2.1. Evaluating Performance on the Task

**Figure 1:**
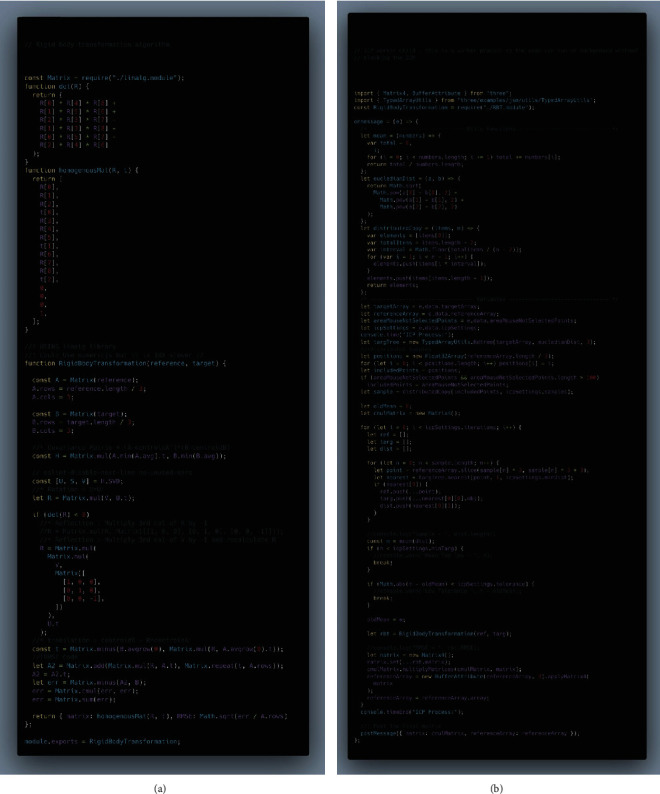
Source code for the dental cavity assessment algorithm: (a) the rigid body transformation algorithm essential for aligning 3D dental scans; (b) the worker code responsible for processing cavity dimensions and evaluating deviations from ideal measurements.

**Figure 2:**
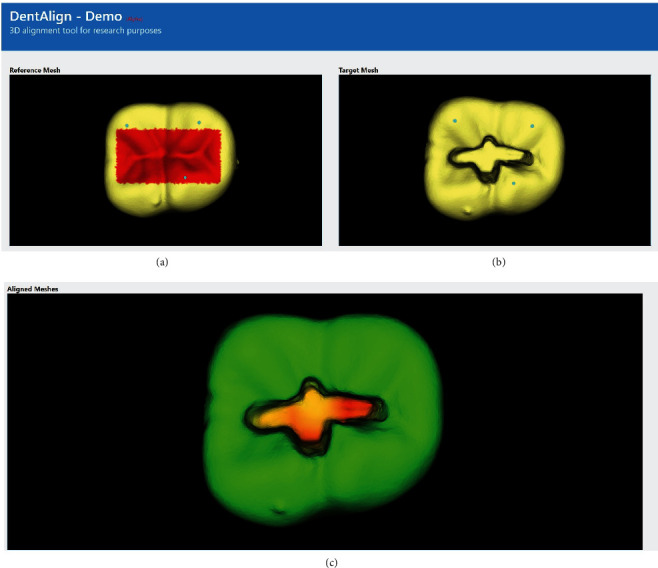
A screenshot from the custom software displaying the aligned prepared tooth (b) and the reference, unprepared tooth model (a). The depth of the preparation is visualized in the corresponding heatmap at (c), allowing for an in-depth comparison with the ideal tooth geometry.

After completing the task, the prepared teeth were collected and optically scanned using Ceramill Map400 (Amann Girrbach). The Standard Tessellation Language (STL) images obtained from the CAD/CAM scanner were then aligned against a reference “unprepared” tooth STL image using a custom software developed by the authors (Figure 1) [13]. The alignment process is carried out by the software using an iterative closest point (ICP) algorithm. The alignment processes the surface information from two sets of 3D point vertices (i.e., reference tooth vs. prepared tooth) to calculate the rigid body transformation using singular value decomposition. The ICP algorithm and the mean squared error of matching two point sets are evaluated by measuring the 3D Euclidean distances between the closest surface points on the two images. Part of the occlusal surface was excluded from the alignment calculation to prevent cavity preparation values from interfering with the initial alignment (Figure 2).

**Figure 3:**
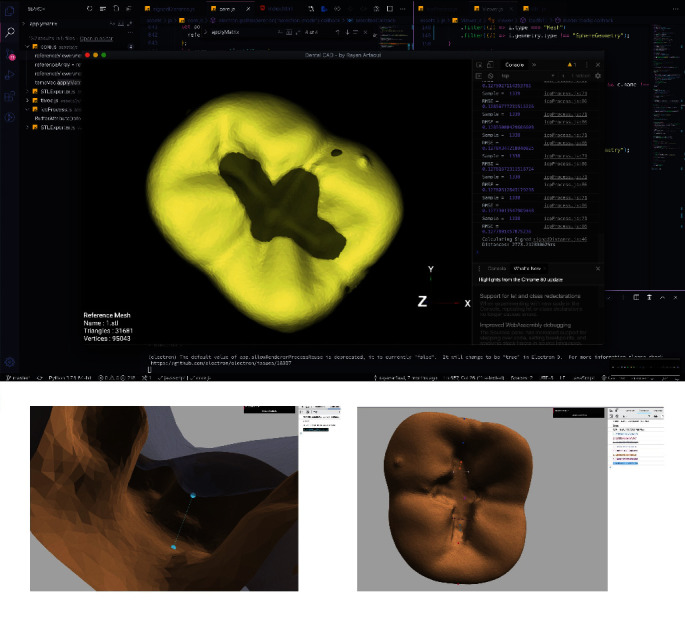
Screenshots from the authors' custom assessment software showcasing the measurement process for cavity preparations. Key dimensions are highlighted: cavity depth readings at mesial, middle, and distal points; isthmus width at mesial and distal points; and remaining tooth structure at mesial and distal marginal ridge areas. These measurements provide comprehensive insight into the quality of the cavity preparation.

Cavity preparations were assessed via a specially programed algorithm software, developed by the authors, using JavaScript language (software is open-source and available at https://cephcad.com/dentalign/) [13]. The software was programed to measure cavity depth, width, and extensions (mesiodistal). Measurements included three depth readings (mesial, middle, and distal), two cavity isthmus width readings (mesial and distal), and two readings for the remaining tooth structure at the marginal ridge area (mesial and distal marginal ridge). The algorithm defined an error as a deviation from the ideal geometry of 0.25 mm and was given an error value of one for each (e.g., an error value of one for a cavity that is 0.25 mm short from, including the mesial pit and an error value of three for a 2 mm wide distal cavity isthmus). The final score for each tooth was therefore calculated as the sum of the number of errors from seven readings (Figure 3).

Cavity preparations were assessed by a panel consisting of three experienced academics specializing in restorative dentistry, selected for their extensive experience and educational roles. Prior to the study, the panel members were oriented to a unified framework of evaluation through a validated assessment sheet, informed by the University of Dundee's criteria for amalgam cavity preparations. This sheet detailed the specific dimensions and characteristics of ideal cavity preparations, ensuring that all evaluators' judgments were grounded in a shared understanding of the assessment criteria.

The panel's calibration process was designed to closely simulate the environment of a standard examination setting, emphasizing the panel's familiarity with the criteria to reflect the natural variance seen in academic evaluations while maintaining the assessment's integrity and relevance. In cases of divergent evaluations, panel members engaged in a consensus discussion, leveraging their collective expertise to determine the final grades. This procedure mirrors the collaborative decision-making typical in academic settings, ensuring fair and comprehensive student evaluations.

### 2.2. Standard Setting and Cutoff Point Determination

To further solidify the reliability of the assessment, the fail mark for cavity preparations was established using the borderline group method, which involved an additional group of 10 experienced dentists. This group independently reviewed a selection of cavity preparations to define the threshold between passing and failing, ultimately identifying a score range of 40–50. The average score within this borderline group was calculated and set as the new cutoff score for pass/fail decisions, which was established at 44. This process provided a clear and empirically grounded benchmark for software comparison, contributing to the study's objective of evaluating the software's effectiveness in a rigorous academic setting.

### 2.3. Statistical Analysis

The grades assigned by both the panel and the software were given out of 100 points. To perform some statistical analyses, these grades were further converted into an alphabetical grading system: A (80–100), B (60–79.99), C (40–59.99), D (20–39.99), and E (0–19.99).

The software's performance in assessing the dental cavity preparations was evaluated using a series of statistical tests. Sensitivity, specificity, positive predictive value (PPV), negative predictive value (NPV), the area under the receiver operating characteristic (ROC) curve, and Cohen's kappa were calculated to understand the software's discriminative ability and its agreement with the panel. To complement these analyses, a precision–recall curve was generated, helping identify an optimal cutoff score for the software.

Additionally, the intraclass correlation coefficient (ICC3k) was computed to measure the degree of agreement between the software and the panel. The ICC3k value offers insight into the consistency of the average of the panel and software's measurements in this specific comparison.

Further insight into the agreement between the panel and the software was sought by conducting a Bland–Altman analysis. This plot-based approach would provide a visual representation of the differences between the panel and the software scores against their averages, thus revealing any potential fixed or proportional bias between the two methods.

Further analysis of the agreement between the panel and the software was conducted using Spearman's rho and Kendall's tau, rank correlation coefficients, measuring the strength and direction of association between the panel and software scores. The associated *p*-values indicated the statistical significance of these correlations.

Lastly, to assess the software's ability to correctly assign grades according to the panel's assessments in the A, B, C, D, and F grading system, a confusion matrix was constructed. Linear and quadratic weighted kappa were calculated based on this matrix to provide more nuanced insights into the agreement, especially considering the ordinal nature of the modified scores.

All statistical analyses were performed using the R software (version 3.6.2; R Foundation for Statistical Computing, Vienna, Austria). For an illustrative representation of the data, heatmaps were generated using Python (Python Software Foundation, version 3.11) with Seaborn 0.11.2 and Matplotlib 3.7.1 packages.

## 3. Results

Table 1 provides a concise comparison of key statistical metrics, including mean, median, mode, standard deviation, variance, and interquartile range (IQR), for both the panel and software assessments of cavity preparations.

**Figure 4:**
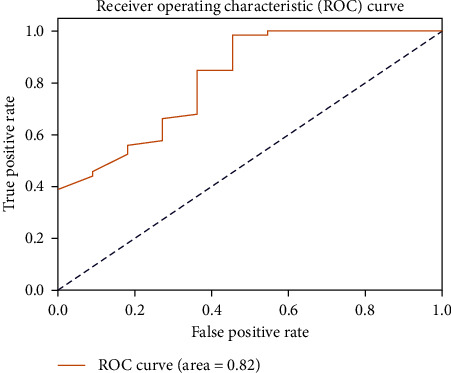
Receiver operating characteristic (ROC) curve for the software's assessment of dental cavity preparations. The curve plots the true positive rate (sensitivity, on the *y*-axis) against the false positive rate (1 − specificity, on the *x*-axis) at various threshold settings. The area under the curve (AUC) represents the software's discriminative ability in distinguishing between correctly and incorrectly prepared cavities. A larger AUC indicates better discriminative performance.

**Figure 5:**
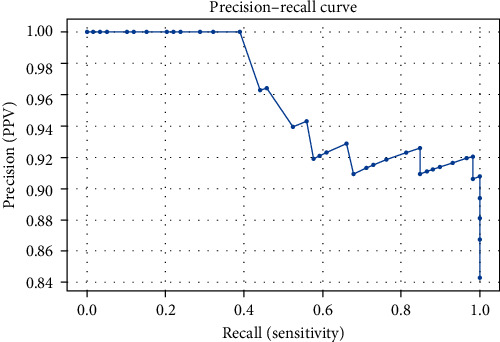
Precision–recall curve illustrating the performance of the software in assessing dental cavity preparations. The curve demonstrates the tradeoff between the precision (*y*-axis) and the recall (*x*-axis) at various threshold settings. The area under the curve (AUC) represents the overall performance of the software in recognizing correct cavity preparations, with a larger AUC indicating better performance.

Table 2 presents the discriminative metrics of the software, encompassing sensitivity, specificity, PPV, NPV, and the area under the ROC curve (AUC). For the ROC curve's visual representation, see Figure 4. The precision–recall tradeoffs at various thresholds are depicted in Figure 5.

**Figure 6:**
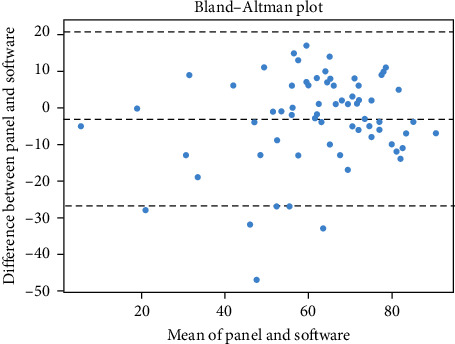
Bland–Altman plot illustrating the agreement between the panel and software scores. The *x*-axis represents the mean of the panel and software scores, and the *y*-axis represents the differences between the two scores. The middle line represents the mean difference (bias), and the outer lines represent the limits of agreement (mean difference ± 1.96 × SD of the difference), encompassing 95% of the differences. The plot reveals any potential fixed or proportional bias between the two assessment methods.

**Figure 7:**
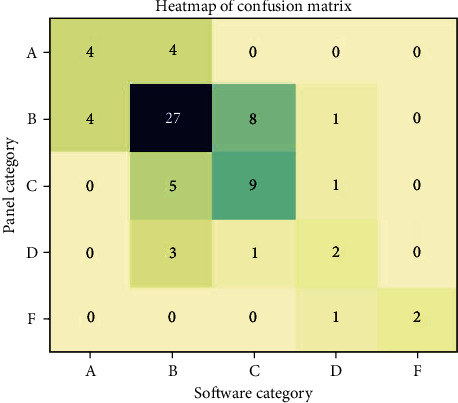
Heatmap of the confusion matrix representing the comparison of grades assigned by the expert panel and the algorithm-based software. The *x*-axis corresponds to the grades assigned by the software, while the *y*-axis corresponds to the grades assigned by the panel. The color intensity reflects the frequency of cases in each grade combination. The diagonal from the top left to the bottom right represents cases where the software and the panel assigned the same grade. Off-diagonal elements represent disagreements.

Table 3 details the agreement metrics between software and panel assessments, offering a precise comparison between the two methods' scores. Further insight into the agreement was gained through a Bland–Altman analysis, visualizing the differences between the panel and software scores against their averages (Figure 6). This approach gave a more detailed picture of the level of agreement between the two methods, revealing any potential fixed or proportional bias. For further analysis on the agreement levels between the panel and software across different grading categories, refer to Figure 7. This heatmap of the confusion matrix visualizes the distribution and frequency of agreement and disagreement on grades, with color intensity highlighting the prevalence of each grade combination.

## 4. Discussion

Our study revealed significant findings regarding the assessment of dental cavity preparations through an innovative integration of algorithm-based software and traditional expert panel evaluations. Key outcomes include the software's high sensitivity (0.98), indicating exceptional accuracy in identifying correct and incorrect cavity preparations, and an impressive intraclass correlation coefficient (ICC3k) of 0.857, demonstrating strong agreement with the expert panel's assessments. These results highlight the software's capability to offer precise, objective measurements of cavity depth, width, and deviations from ideal geometry, closely aligning with the experienced academics' validated assessments in operative dentistry education.

The decision to use a mandibular third molar in our study was primarily to minimize the inherent biases associated with expert judgment. Opting for a tooth that is less frequently encountered in routine evaluations allowed us to create a level playing field for both human and software assessments. This approach was particularly important to reduce familiarity bias, ensuring that the evaluations were based solely on the objective criteria set forth by the study, rather than prior experience or subjective judgment.

The software tended to assign slightly higher scores on average than the panel, which might reflect a more lenient evaluation by the algorithm, focusing on quantifiable aspects of cavity preparations and possibly overlooking some subjective criteria valued by experts [14, 15]. Despite this leniency, the variation in scores from both the software and the panel underscored a moderate performance level overall, pointing to the complex nature of operative dentistry and the diversity in technique. Such variability is indicative of the challenges inherent in standardizing assessments in a field where individual technique plays a significant role.

A noteworthy aspect of the software's performance was its high sensitivity and precision, indicating significant accuracy in distinguishing between successful and unsuccessful cavity preparations. Although there was a slight inclination toward false-positive errors, the high PPV and NPV suggested that the software-assigned grades were largely reliable indicators of performance.

The software's discriminative ability, as illustrated by the ROC curve, was good, and the AUC further reinforced this result. The precision–recall curve suggested that a balance between the precision and recall of the software can be achieved at various thresholds, making it a valuable tool for real-world assessment scenarios.

The analysis of agreement between the software and the panel yielded intriguing results. A high level of agreement, as reflected by the ICC3k, suggests that the software and panel's assessments were largely consistent. This outcome gives promising evidence for the algorithm's capacity to replicate human judgment in a standardized and unbiased manner. Bland–Altman analysis, Cohen's kappa, and correlation coefficients further supported this finding by demonstrating a strong agreement and positive correlation between the two methods.

It is worth noting that the software agreed more on the lower-performing samples than on the higher-performing ones. This observation implies that while the software can effectively identify underperformance, it may not be as proficient at recognizing top performers, possibly due to the intricacies of optimal preparations that are more readily appreciated by human experts. However, arguably, identifying lower-performing students can be more critical for educational purposes, as these students would greatly benefit from immediate intervention and feedback to improve their skills [16]. Early identification of struggling students has been evidenced to result in significantly better outcomes in academic performance and skill development [17, 18]. Therefore, our software's tendency to reliably detect underperformance is not necessarily a drawback but rather a valuable feature for effective pedagogical practice.

The analysis of the ordinal agreement, demonstrated through the linear weighted kappa of 0.51 and quadratic weighted kappa of 0.66, indicated a moderate-to-substantial agreement when considering the grades' ordinal nature. These findings suggest that while the software can effectively categorize performance into grades, it still requires refinement to match the panel's grading precision accurately. This analysis underscores the need for further development of the software to enhance its capacity for evaluating dental cavity preparations with precision akin to that of expert evaluators.

Interestingly, our findings resonate with those of related projects (i.e., E4D Compare Software and PrepCheck) that noted growing affinity toward technology-facilitated evaluations, particularly in dental education [3, 19, 20, 21]. Dental academics' favorable reception of these technologies can be attributed to its perceived objectivity and consistency, attributes that align with the features of our algorithm-based software. Hence, our study further emphasizes the potentially transformative impact of such tools on the evaluation process within dental education.

This inclination of academics toward technological tools for evaluation also addresses the pervasive issue of skepticism toward subjective judgment. By providing a standardized, unbiased evaluation, our software could alleviate these concerns and circumvent potential disputes over grading legitimacy.

Digital assessment limitations, highlighted previously in the literature [19], mirror our own and point to areas for improvement, especially when it comes to cavity convergence, divergence, and outline form. It underlines the need for further validation of the software's accuracy, an aspect that our study also acknowledges. The software is an invaluable tool for assessing the primary parameters of cavity preparations; however, it still requires further development to thoroughly evaluate other significant factors in dental preparations.

In our study, both precision, defined as the measure of how often the software's positive predictions are correct, and sensitivity, indicating how well the software identifies actual positives, were remarkable [22, 23]. These robust metrics indicate that the software could potentially function as a standalone assessment tool, capable of delivering consistent and accurate evaluations without the need for an accompanying expert panel. This independence aligns with previous findings in dental education, where digital assessment tools have shown promise in achieving positive outcomes across differing methodologies [5, 12, 20, 24, 25].

This does not negate the value of an expert panel's insights and experience, but rather, it proposes an efficient alternative for instances where immediate, objective, and scalable assessment is advantageous. Our findings are corroborated by those of another study, which reported that excellent repeatability of digital evaluations does not necessarily equate to valid grading [24]. As such, striking a balance between human expertise and the software's precision and sensitivity could provide comprehensive, accurate, and efficient assessments, ultimately improving the quality of dental education.

Our findings underscore the value of algorithm-based software in dental cavity preparation assessments, offering a valuable supplement to traditional evaluation methods. There is room for improvement in the software's ability to recognize top-tier preparations, ensuring alignment with expert panel assessments. Such advancements will enhance the software's reliability and utility in operative dentistry education. Additionally, the software's capacity for customization and adaptability underlines its potential for wider educational use. By allowing for the personalization of comparison criteria and standardizing teaching methodologies, our software paves the way for its further development, promising a more targeted and comprehensive feedback mechanism.

One notable limitation of our study lies in its focus on cavity preparations using a mandibular third molar and specifically for amalgam restorations. This approach, while providing a rigorous test of precision, may not fully represent the broad spectrum of clinical scenarios encountered in contemporary dental practice. As the dental field continues to evolve with a shift toward composite restorations and inlays, moving away from amalgam, the specific choice of tooth and restoration material in this study may limit the generalizability of our findings. However, we emphasize the software's capability to adapt to a variety of restorative procedures, including inlay preparations and composite restorations, provided these are defined with clear evaluative criteria. This adaptability suggests that while our study used amalgam restorations for its initial assessment, the software's utility is not confined to this material alone, offering potential applicability across different restorative techniques. Additionally, the study was conducted within a controlled academic setting, which might not fully capture the complexities and variations of real-world clinical environments. Future studies could expand the scope by incorporating a wider range of teeth and restoration materials, including composites, to better reflect current dental practices and materials. Moreover, integrating the software's assessment capabilities into a clinical setting could provide insights into its practical utility and areas for further refinement.

## 5. Conclusion

The outcomes of this investigation underscore the algorithm-based software's efficacy in closely aligning with the assessments of well-trained and calibrated dental educators. This alignment reaffirms the high standards of teaching and evaluation upheld by experienced educators in the field of dentistry. While our findings do not suggest the software is superior to human evaluation, they underscore its potential as a complementary tool that enhances traditional assessment methods.

By integrating the objectivity and consistency of algorithmic assessment with the comprehensive understanding and personalized feedback from skilled educators, this software can enrich the evaluative process, making it more robust and comprehensive. Thus, the study contributes to the evolving landscape of dental education by illustrating how technology can support and augment the expert judgment of dental educators, rather than replace it. Future research may focus on quantifying the added value of such technologies in terms of efficiency, bias reduction, and educational outcomes to further define their role in dental education.

## Figures and Tables

**Table 1:** Summary of statistical measures for panel and software scores.

Metric	Panel scores	Software scores
Mean	60.91	64.1
Median	67.5	66.5
Mode	69	69
Standard deviation	18.24	16.1
Variance	332.76	259.32
Minimum score	3	8
Maximum score	87	94
Range	84	86
1st quartile (Q1)	53.5	56.25
3rd quartile (Q3)	72.75	74.0
Interquartile range (IQR)	19.25	17.75

**Table 2:** Discriminative metrics of the software.

Metric	Score
Sensitivity	0.98
Specificity	0.55
Positive predictive value (PPV)	0.92
Negative predictive value (NPV)	0.86
Area under the ROC curve (AUC)	0.82

**Table 3:** Agreement metrics between software and panel assessment.

Metric	Value	Note
ICC3k	0.857	High level of agreement; 95% CI = [0.77, 0.91], *p* < 0.01
Cohen's kappa	0.62	Substantial agreement
Spearman's rho	0.73	Strong positive correlation; *p* < 0.01
Kendall's tau	0.56	Strong positive correlation; *p* < 0.01
Agreement on worst 5%	80.00%	—
Agreement on top 20%	65.00%	—
Linear weighted kappa	0.51	Moderate agreement
Quadratic weighted kappa	0.66	Substantial agreement

*Note*. The intraclass correlation coefficient (ICC3k) measures the overall agreement between the software and the panel. Cohen's kappa, Spearman's rho, and Kendall's tau indicate the consistency in ranking and grading between the two methods. The linear and quadratic weighted kappa values assess the degree of agreement, factoring in the severity of disagreements.

## Data Availability

The data that support the findings of this study are available on request from the corresponding author.
